# Estimating population size using single‐nucleotide polymorphism‐based pedigree data

**DOI:** 10.1002/ece3.2076

**Published:** 2016-04-06

**Authors:** Robert Spitzer, Anita J. Norman, Michael Schneider, Göran Spong

**Affiliations:** ^1^ Wildlife Ecology GroupDepartment of Wildlife, Fish and Environmental StudiesSwedish University of Agricultural SciencesSE‐901 83UmeåSweden; ^2^ Molecular Ecology GroupDepartment of Wildlife, Fish and Environmental StudiesSwedish University of Agricultural SciencesSE‐901 83UmeåSweden; ^3^ Västerbotten County AdministrationSE‐901 86UmeåSweden; ^4^ Forestry and Environmental ResourcesCollege of Natural ResourcesNorth Carolina State UniversityRaleigh27695North Carolina

**Keywords:** Brown bear, noninvasive sampling, pedigree reconstruction, population estimate, rarefaction, single‐nucleotide polymorphism, SNP

## Abstract

Reliable population estimates are an important aspect of sustainable wildlife management and conservation but can be difficult to obtain for rare and elusive species. Here, we test a new census method based on pedigree reconstruction recently developed by Creel and Rosenblatt (2013). Using a panel of 96 single‐nucleotide polymorphisms (SNPs), we genotyped fecal samples from two Swedish brown bear populations for pedigree reconstruction. Based on 433 genotypes from central Sweden (CS) and 265 from northern Sweden (NS), the population estimates (*N *=* *630 for CS,* N *=* *408 for NS) fell within the 95% CI of the official estimates. The precision and accuracy improved with increasing sampling intensity. Like genetic capture–mark–recapture methods, this method can be applied to data from a single sampling session. Pedigree reconstruction combined with noninvasive genetic sampling may thus augment population estimates, particularly for rare and elusive species for which sampling may be challenging.

## Introduction

Estimates of the population size and its fluctuations are often fundamental for understanding ecological, behavioral, or genetic processes (Ojaveer et al. 2004; Dochtermann and Peacock 2013; Valderrama et al. 2013) and practically indispensable for management and conservation (Katzner et al. 2011). This includes estimates of both effective and true population size, where the former is usually based on genetic data and models, while the latter typically use some form of census data, sometimes genetic. For example, such population size and trend estimates help identify particular factors that drive population dynamics and are hence critical for modeling the future of a population under different management scenarios (Lewellen and Vessey 1998). Moreover, estimates of true population size and trend are the basis for adaptive harvest quotas (Wilson and Delahay 2001) as well as identifying populations under threat of becoming endangered or extinct (Vié et al. 2009). However, reliable estimates are difficult to obtain. This is especially true for rare and elusive species, which are frequently of high conservation concern (Rolland et al. 2011). Large carnivores are no exception to this (Kindberg et al. 2009; Creel and Rosenblatt 2013) as they are generally solitary and cryptic, in addition to occurring at low densities and across large home ranges. For several ecological, economic, and societal reasons, large carnivores in particular receive a disproportional amount of attention from research, conservation, and management. For example, carnivores may strongly affect the ecosystems they occupy, by changing the behavior of other carnivores and by direct and indirect effects on prey, which can lead to downstream effects on primary production (Creel et al. 2007). In some areas, carnivores pose a threat to humans or come into direct conflict with human husbandry practices causing economic losses. For these, and more reasons, a range of remote or noninvasive methods have therefore been employed to study carnivores (Jackson et al. 2006; Kojola et al. 2014). An increasingly popular and cost‐efficient approach is to use noninvasive genetic sampling for the assessment of the number of individuals in a population (e.g., Mowry et al. 2011; Sugimoto et al. 2012; Stansbury et al. 2014) often by collecting fecal samples during other management activities or by citizen volunteers (e.g., Kindberg et al. 2011).

Genetic data may also be used to assess a population's effective population size, an important parameter especially for small populations at risk of inbreeding or genetic drift. The framework of population genetics provides several way of inferring the effective population size, but the estimators suffer from being slow to respond to recent events, instead showing historic averages (Palsboll et al. 2013). To obtain more contemporary estimates, genetic data can be also used to derive demographic data used for calculating the current effective population size (e.g., Creel 2002). For many studies in ecology, however, the actual population size is a more important parameter to know than the underlying effective population size. Also in conservation, much focus has been placed on the effective population size. Yet, as pointed out by Lande (1988), the drivers of extinction are primarily habitat loss and overharvest, not lack of genetic variation. So while it is informative to know the effective population size, once its relationship to the actual population size has been determined, its continuous monitoring may be less important than knowing the actual population size. This is equally true for critically endangered populations at the verge of extinction as for larger populations not acutely threatened. Here, the actual population size is typically what management operates on when setting targets for quotas, dispersal events, or the population size and distribution.

Most statistical methods for estimating population size rely on multiple sampling events, known as capture–mark–recapture (CMR) techniques which are comprehensively discussed by Krebs (1999) and Sutherland (2006). A distinct disadvantage of classical CMR methods lies in the circumstance that the physical capture, particularly of large predators, is often impractical, costly, and potentially harmful to both sides (Mowat et al. 1994; Logan et al. 1999; Muñoz‐Igualada et al. 2008). In addition, differences in catchability resulting from trap‐shy or trap‐happy individuals could introduce systematic trapping bias. Such differences in personality traits (Sih and Bell 2008) have been documented for many species, including badgers (Tuyttens et al. 1999), stoats (King et al. 2003), or rabbits (Sunnucks 1998).

Newer methods, such as camera trapping, have largely made classical CMR approaches obsolete in studies of large animals. But many cameras are needed to reach reasonable detection probabilities (and cameras are sometimes removed or destroyed by humans or other animals). Even more problematic is that relatively few species are reliably individually identifiable from photographs. In contrast, an individual's genotype is a unique and permanent mark. Noninvasively collected DNA samples (e.g., from feces or hair) in combination with molecular techniques offer another noninvasive alternative (Kohn and Wayne 1997; Taberlet et al. 1999; Waits and Paetkau 2005; Swenson et al. 2011). In direct genetic census methods, the genotype simply becomes a “molecular tag” (Schwartz et al. 2007) which replaces traditional means of identification like earmarks or leg bands. Genotypes can thus be used as molecular tags in a CMR framework. But genetic data contain more information than just individual genotypes, such as information on pedigree structures in the population. From such information, unsampled individuals could potentially be inferred by their genetic fingerprint and included into the population estimates.

In 2013, Creel and Rosenblatt suggested a new, pedigree‐based estimator for total population size. They evaluated the performance of their method through simulations parameterized with demographic data of African lions (*Panthera leo*) from Zambia. The method, henceforth referred to as the Creel–Rosenblatt estimator (CRE), incorporates the sum of sampled individuals (*N*_s_), number of breeders (*B*_s_), number of individuals inferred from pedigree reconstruction (*N*_in_), and the estimated number of individuals that did not breed nor were sampled (rendering them invisible to pedigree reconstruction) into the population estimate. As such, it purports to increase the precision of genetically based population estimates.

As other genetically based CMR methods, the CRE requires only one sampling event (although multiple sampling events are also possible). This makes it a useful extension to the suite of tools available to estimate population sizes under circumstances where repeated sampling is difficult. In addition to being a novel approach for estimating population size, pedigree reconstruction can be used to investigate population structure (Calboli et al. 2008; Pemberton 2008), mating behavior (Pemberton et al. 1992), or dispersal (Norman and Spong 2015). The ideal genetic marker for pedigree reconstruction should provide high genomic resolution and be geared toward providing reliable relatedness estimates (Creel and Rosenblatt 2013). Single‐nucleotide polymorphisms (SNPs) have proven to be a powerful tool for studying genetic variation in populations (Brumfield et al. 2003; Morin et al. 2004). Compared with microsatellites, another type of frequently used genetic marker, SNP, offers lower error rates from mistyping and allelic dropout (Morin and McCarthy 2007; Norman et al. 2013). They are also reproducible across laboratories and are cheaper, allowing for higher genomic resolution within a given economic frame (Anderson and Garza 2006). Because only short intact sequences, typically 50–70 bp, of DNA are required for successful amplification, SNPs are especially suitable when working with degraded DNA, as is usually the case with noninvasively obtained samples (Morin et al. 2004).

**Figure 1:**
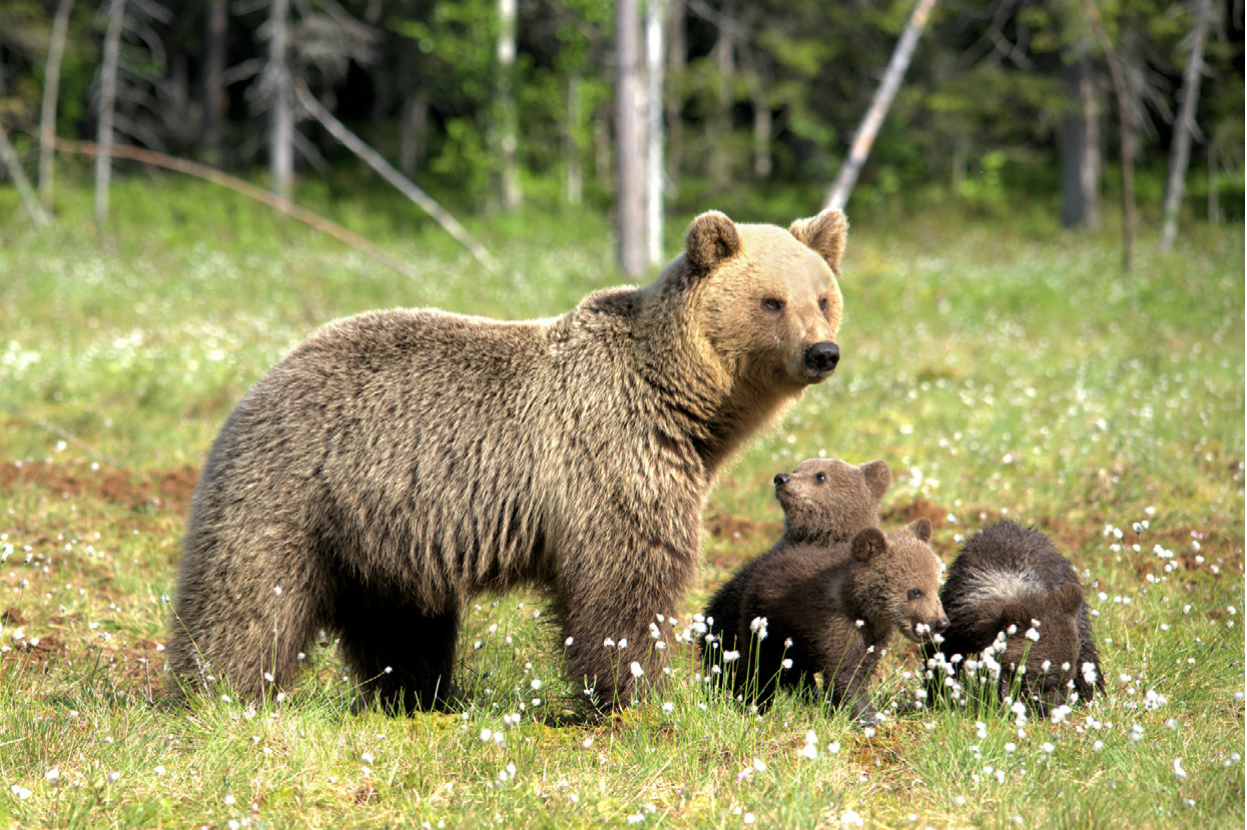
A female Scandinavian brown bear with cubs. Source: Nyhetsbyrån.

Here, we use a panel of 96 SNPs recently developed for studying relatedness in the Scandinavian brown bear (*Ursus arctos,* Fig. 1) population (Norman et al. 2013). We reconstructed pedigrees based on hunter‐collected feces and apply the CRE method to estimate the size of the brown bear populations in the Swedish counties of Dalarna, Gävleborg, and Västerbotten. Already existing population estimates for the brown bear in these areas (Kindberg and Swenson 2013, 2015) provide us with a benchmark that can be used to empirically assess the performance of the CRE outside of a simulation environment making this study system appropriate. For further comparison, we also performed rarefaction analyses to estimate population size. This constitutes the first time that the estimator is applied to empirical data, as we were unable to find any reference describing the application of this method in the Web of Science^™^ publication database, with the last search completed on 17 November 2015.

## Materials and Methods

### Study area and sample collection

**Figure 2:**
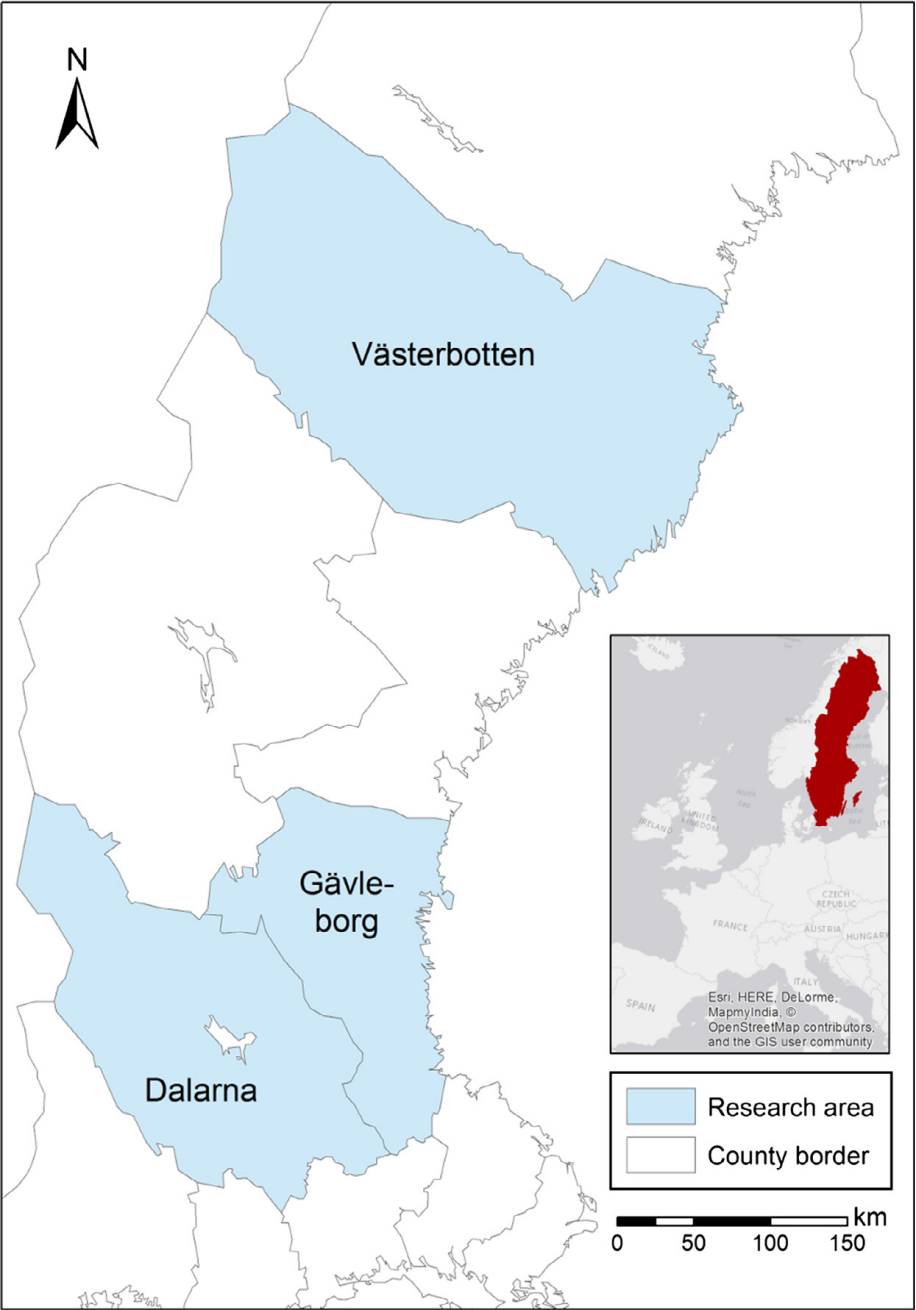
Map showing the location of the study areas (blue) within Sweden (red).

The two study areas in central and northern Sweden encompassed the Swedish counties of Dalarna and Gävleborg (ca. 46,300 km²) and Västerbotten (ca. 55,200 km²), respectively. To the west, these areas are delimited by the Scandinavian mountain range and to the east by the Baltic Sea (Fig. 2). The southern border of Dalarna–Gävleborg also demarcates the approximate southern limit of the brown bear distribution in Sweden. Dalarna–Gävleborg is home to an estimated number of 793 bears, 95% CI [621, 1179] (Kindberg and Swenson 2013). In 2014, the Västerbotten population was estimated to be 362 bears, 95% CI [310, 459] (Kindberg and Swenson 2015). Studies of maternally inherited mitochondrial DNA (mtDNA) have shown that brown bears in Sweden belong to two genetically distinct lineages with approximately 7% differentiation between them (Taberlet and Bouvet 1994). The western lineage, found in south‐central Sweden, originated from the Iberian refugium during the last ice age (today's France and Spain), whereas the eastern lineage, found throughout northern Sweden, can be traced to Karelia in Russia (Taberlet and Bouvet 1994). At present, the two lineages remain largely separated around a well‐documented contact zone at the height of Östersund in central Sweden (Taberlet et al. 1995). Population monitoring could be especially important in case of the western mtDNA haplotype (southern population) which is only found in Europe, whereas the eastern haplotype is also prevalent in Asia and North America (Waits et al. 2000; Saarma et al. 2007; Korsten et al. 2009; Hirata et al. 2013).

Fecal samples were collected by volunteers, predominantly moose (*Alces alces*)‐hunters, following the protocol of Bellemain et al. (2005) and Kindberg et al. (2011) during the periods of August–October 2012 in Dalarna–Gävleborg and August–December 2014 in Västerbotten.

Volunteers recorded collection date and coordinates of the sample location and mailed this information together with their samples to the county administrations (in the case of the Dalarna–Gävleborg collection) or in Västerbotten directly to the Molecular Ecology Group at the Swedish University of Agricultural Sciences (SLU) in Umeå. Upon arrival samples were stored in 70% ethanol solution at −20°C as recommended by Frantzen et al. (1998).

### Molecular analysis

DNA extraction from the Dalarna–Gävleborg samples was carried out by Bioforsk, Norway (Hagen and Aarnes 2013), following procedures described by Schregel et al. (2012). In Västerbotten, DNA extraction was performed at SLU using a QIAsymphony SP (Qiagen, Hilden, Germany) robot according to the manufacturer's instructions.

SNPs were genotyped on a Fluidigm Biomark^™^ (Fluidigm Corporation, San Francisco, USA) using the 96 SNP panel developed by Norman et al. (2013). Since its first publication, the panel has undergone slight modifications (e.g., two linked SNPs were substituted with Y‐chromosome SNPs) and now consists of 85 autosomal SNPs, four mtDNA SNPs as well as four Y‐chromosome and three X‐chromosome markers for sex determination (Norman and Spong 2015). Each run included negative controls with water in place of DNA. The genotype clusters assigned by the Biomark software were manually screened, and loci of questionable cluster affiliation were invalidated and removed from subsequent analyses. Species and sex were assigned according to the following criteria:

- bear* *=* *mtDNA SNP calls ≥3
- male* *=* *Y‐chromosome SNP calls ≥3
- female* *=* *Y‐chromosome SNP calls* *=* *0 and X‐chromosome SNP calls ≥2

The above criteria were designed to avoid possible misidentification of poorly amplified male samples as females. In males, Y and X markers occur in equal proportion. The requirement that at least two of three X markers had to amplify for samples to be called female makes it extremely unlikely that such a sample was a male that had not amplified for the Y markers. As we only included samples that had amplified for more than 70 loci, the risk of having none of four Y markers but two of the X amplify for a male is 1 × 10^−4^.

### Pedigree reconstruction

To reconstruct pedigrees, we used FRANz software version 2.0.0 (Riester et al. 2009) which uses Markov Chain Monte Carlo (MCMC) simulation for estimating the statistical confidence of parentage inference. The software requires specifying an approximate maximum number of females and males (Nfmax and Nmmax) to avoid an empty pedigree due to convergence of the Markov Chain to a very high number of individuals (Riester et al. 2009). We used the estimates from rarefaction analysis and the sex ratio present in the genotyped samples to set Nfmax/Nmmax to 538/419 (Dalarna–Gävleborg) and 249/239 (Västerbotten), respectively. Typing errors were empirically determined to 1.538 × 10^−4^ for Dalarna–Gävleborg and 0.01 for Västerbotten. The error rates from the two areas differ. This is because samples from Dalarna–Gävleborg held the best available quality extract from each individual successfully genotyped with microsatellites at Bioforsk, whereas the error rate for the Västerbotten samples includes all samples that passed the amplification threshold for SNP genotyping. As microsatellite genotyping requires much higher quality DNA, the error rate of such samples becomes much lower. The maximum likelihood pedigrees produced by FRANz identify the putative sire and dam of sampled individuals. We further verified the FRANz reconstructed pedigrees by calculating the Lynch–Ritland relatedness coefficient (*r*) (Lynch and Ritland 1999) for all identified parent–offspring (PO) and full‐sibling (FS) pairs using COANCESTRY version 1.0.1.2. (Wang 2011). We chose the Lynch–Ritland relatedness coefficient because it has been found to have the lowest rate of misclassification and lower overall variance compared to other pairwise relatedness estimators (Stone and Björklund 2001; Csillery et al. 2006).

### Population estimates

Rarefaction, also referred to as accumulation‐curve method, has traditionally been used to estimate species diversity in an area by plotting the cumulative number of newly recorded species against the total number sampled (Colwell and Coddington 1994). The same underlying logic can be applied for estimating population size by substituting the species count with the number of unique individuals/genotypes. As suggested by Kohn et al. (1999), a curve defined by the equation *y *=* ax/(b+x)* was fitted to our data. In this model, *y* equals the number of unique genotypes, *x* corresponds to the number of samples (genotyped feces), *b* is the rate of decline in the slope, and the asymptote *a* represents the estimated population size (Bellemain et al. 2005). We calculated the parameters *a* and *b* through nonlinear iterative regression using the statistical software package JMP Pro version 11.0.0 (SAS Institute). To account for the variance caused by the order in which samples are drawn, we repeated this process 100 times with random iterations of the genotype sampling order and used the mean of the resulting asymptotes as the rarefaction population estimate.

For the pedigree‐based population estimates, we followed the recommendations by Creel and Rosenblatt (2013) and specified the number of individuals sampled (*N*_s_) as the number of individual genotypes, known breeders (*B*_s_) as those individuals who had progeny in the pedigree and inferred individuals (*N*_in_) as the missing parent in known parent–offspring dyads. However, assuming that each missing parent in the dyads constitutes a new individual would most likely cause an overestimation because brown bear males are known to mate with several females and vice versa (Steyaert et al. 2012). For example, an inferred sire may be the missing father in more than one of the mother–offspring dyads. Therefore, we used the improbable scenario in which the number of inferred individuals (*N*_in_) equals the number of dyads in the pedigree only for approximating an upper bound of the population estimate. For a more realistic estimate that accounts for multiple parentages, we first screened all parent–offspring dyads in the pedigree for individuals with several offspring. If pairwise comparisons of the Lynch–Ritland relatedness suggested full‐siblings (*r* ~ 0.5) among those offspring, we inferred only one new individual (the missing parent) from these dyads. For the remaining cases, we used a different approach where we assumed that the likelihood of sampling each sex was equal: We determined the ratio of all the known individual dams to the known individual sires in the pedigree and then used this ratio to infer the missing counterparts from the individual dams and sires in the pedigree dyads. In this way, the ratio of dams to sires with the inferred individuals included remains the same as it was in the original pedigree.

Another problem pointed out by Creel and Rosenblatt (2013) is the circumstance that there is no way to ascertain how many of the inferred individuals are actually still alive at the time of the estimate. To account for mortality among inferred individuals, we assumed them to be at the typical breeding age of ~5 years (Swenson et al. 2001) and applied the age‐specific annual mortality rates as reported in Nilsson (2013) of 7.2% to inferred dams and 11.6% to sires, respectively.

Finally, we assessed the accuracy of the CRE results by comparing them to the official population estimates (Kindberg and Swenson 2013, 2015) and to the results of rarefaction analysis. Because there is currently no method to assign confidence limits to the CRE population estimates, we estimated an upper and a lower bound. For the lower bound, we simply used the count of sampled genotypes. For the upper bound, we treated *N*_in_ as equal to the number of dyads in the pedigree and assumed zero mortality among the inferred individuals.

To test the performance of the CRE at different sampling intensities, we used the data from Västerbotten due to the higher sampling coverage (approximately 73% of the population included in the sample) compared to only 55% in Dalarna–Gävleborg. Varying the sampling intensity from 10% to 60% of the official population estimate, we applied the CRE to ten replicates of samples randomly drawn in correspondence with each sampling intensity level.

Not having 100% of the population included in the sample is a common limitation in studies based on field data, but validation of simulation results using empirical data may still reveal strengths and weaknesses.

We assumed that the pedigree would become less complete (contain fewer parent–offspring pairs) the further away the sampling occurred from the core sampling frame. This is because breeding individuals in peripheral areas might have moved beyond the borders of the sampling area and therefore may have been missed during the sample collection. In both areas, Dalarna–Gävleborg and Västerbotten, the only true population border is the Baltic Sea to the east. To the north and the south, bears occur beyond the borders of the sampling areas. Most interesting is the border to the west, formed by the Scandinavian mountain range. Because mountain terrain can be difficult to access and because moose hunting is of lower intensity at higher altitudes, the sampling effort by volunteers was lower there than in other areas. If the pedigrees were to show similar levels of incompleteness along the western border compared with the “open” borders to the north and the south, it could indicate that bears in the mountains were missed in the sampling. If this were the case, it would lead to an underestimation of the population size. If, on the other hand, the mountains form a true border like the Baltic Sea, then the pedigree should be equally complete in both these locations.

From the coordinate data that were provided along with the fecal samples, the median centers of all known locations for an individual were calculated using R (R Development Core Team, 2008). We considered the median to be less biased than the mean because of its lower sensitivity to outliers. Inferring home ranges from the locations of fecal samples is prone to errors but Bellemain et al. (2005) reported that the majority of fecal sites fall inside the home range or within 10 km of it. We determined the center point of the sampling area as the median center of all individual locations using the GIS package ArcMap version 10.2.2 (ESRI, 2014). We then sampled the individuals closest to the center point and the four borders (north, south, east, and west), respectively, at sample sizes of *n *=* *100 for Dalarna–Gävleborg and *n *=* *70 in Västerbotten. The number of samples for Västerbotten had to be lower to avoid overlap because fewer individuals in total were available to sample from. In a second step, we also sampled males and females separately (Dalarna–Gävleborg, *n *=* *50; Västerbotten, *n *=* *30) to investigate whether there are detectable differences between mother–daughter and father–son dyads.

To test for differences in the completeness of the pedigree, we used Pearson's chi‐square test for homogeneity of proportions with the proportions corresponding to the number of parent–offspring pairs in the pedigree per number of sampled individuals. To further test whether there is a spatial effect on parent–offspring pairs in sex‐separated pedigrees, we also sampled males and females randomly across the whole sampling area (Dalarna–Gävleborg, *n *=* *50; Västerbotten, *n *=* *30).

## Results

We successfully genotyped 433 individuals (243 females, 190 males) for Dalarna–Gävleborg and 265 individuals (136 females, 129 males) for Västerbotten. Rarefaction analysis was based on 873 samples from Dalarna–Gävleborg and 677 from Västerbotten. The maximum frequency at which an individual occurred in the sample was 19 for Dalarna–Gävleborg and 16 for Västerbotten, respectively. The amplitudes of the curves fitted to the rarefaction data suggested population sizes of *N *=* *895 (Dalarna–Gävleborg) and *N *=* *484 (Västerbotten).

**Figure 3:**
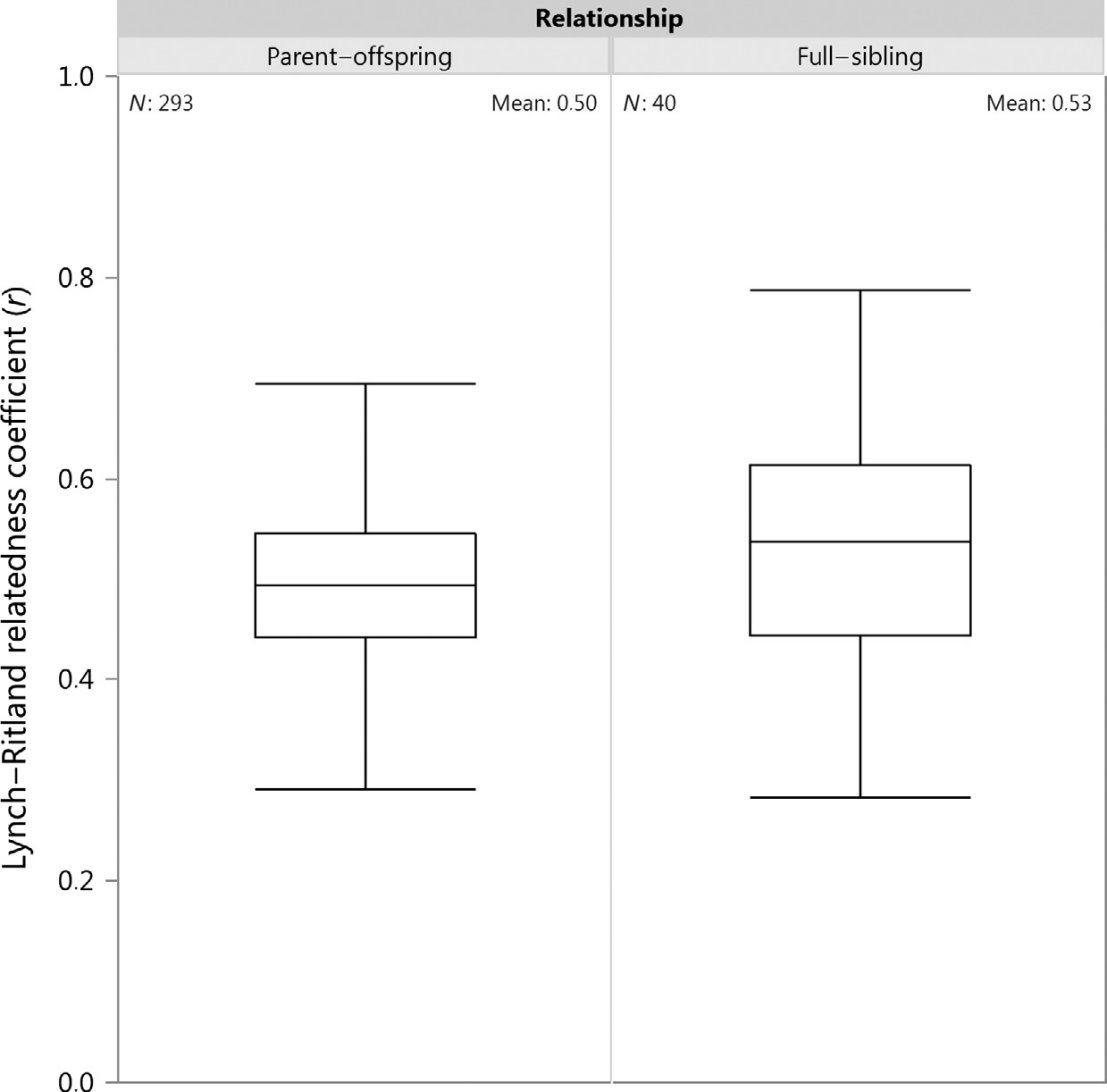
First‐order relatives in the reconstructed pedigrees correspond well with the expected value (*r *=* *0.5) for the Lynch–Ritland relatedness coefficient.

Table 1 summarizes the results of the FRANz reconstructed pedigrees. The proportions of breeders in both samples (0.37 Dalarna–Gävleborg, 0.42 Västerbotten) are not significantly different (*z *=* *−1.42, *P *=* *0.16), and the ratios of dams to sires are also very similar. As shown in Figure 3, the mean pairwise Lynch–Ritland relatedness coefficient (*r*) for PO dyads and FS pairs did not significantly differ from the expected value of *r *=* *0.5 for first‐order relatives (PO: *t*(292)* *=* *0.33, *P *=* *0.74; FS: *t*(39)* *=* *1.60, *P *=* *0.12) which corroborates the reconstructed pedigrees.

**Table 1:** Key characteristics of the reconstructed pedigrees showing the number of individuals with both parents identified (triads), one parent identified (dyads) or no identified parent. *N*_s_ denotes the number of directly sampled individuals (number of genotypes), and *B*_s_ corresponds to known breeders (individuals with at least one offspring in the pedigree)

	Dalarna–Gävleborg	Västerbotten
*N*_s_	433	265
Number of triads	65	37
Number of dyads	170	123
Number with “no parent”	198	105
*B*_s_	159	112
Ratio of dams: sires	1.30	1.20

**Figure 4:**
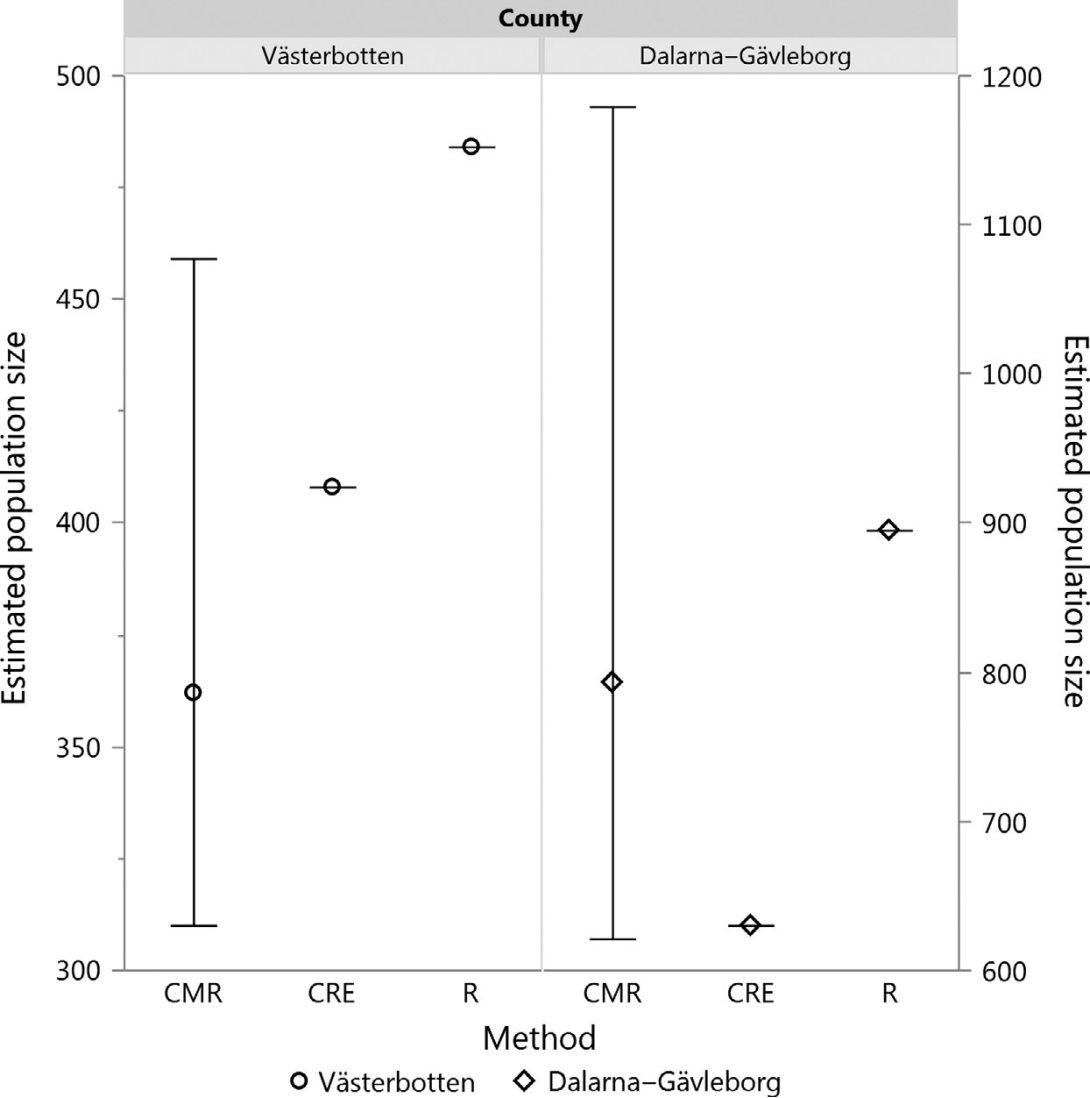
The pedigree reconstruction‐based population estimates of the Creel–Rosenblatt estimator (CRE) fall within the 95% confidence limits of the official estimates based on multiple capture–mark–recapture (CMR) techniques. Rarefaction analysis (R) using the extrapolation model suggested by Kohn et al. (1999) resulted in higher estimates.

In Dalarna–Gävleborg, we inferred six sires and four dams directly from full‐sibling relationships among the known parent–offspring dyads. An additional 52 sires and 65 dams were inferred using the dam to sire ratio approach. After correcting for mortality, the total number of inferred individuals (*N*_in_) (i.e., those missing from the pedigree) was 115. For Västerbotten, screening the parent–offspring dyads for full‐siblings yielded four sires and three dams, whereas the ratio method suggested a further 41 sires and 45 dams resulting in *N*_in_ **=* *85 after mortality correction. Therefore, applying CRE to these numbers (*N*_s_ and *B*_s_ from Table 1 and *N*_in_) resulted in estimated population sizes of *N *=* *630 for Dalarna–Gävleborg and *N *=* *408 for Västerbotten. Comparison with official bear population estimates shows that both CRE results fall within the 95% CI of official estimates (Fig. 4). Using the genotype count as a measure for minimum population size and number of dyads for the estimate of *N*_in_ under the assumption of no mortality, the lower and upper bounds for the CRE correspond to 433 and 728 in Dalarna–Gävleborg and to 265 and 476 in Västerbotten.

**Figure 5:**
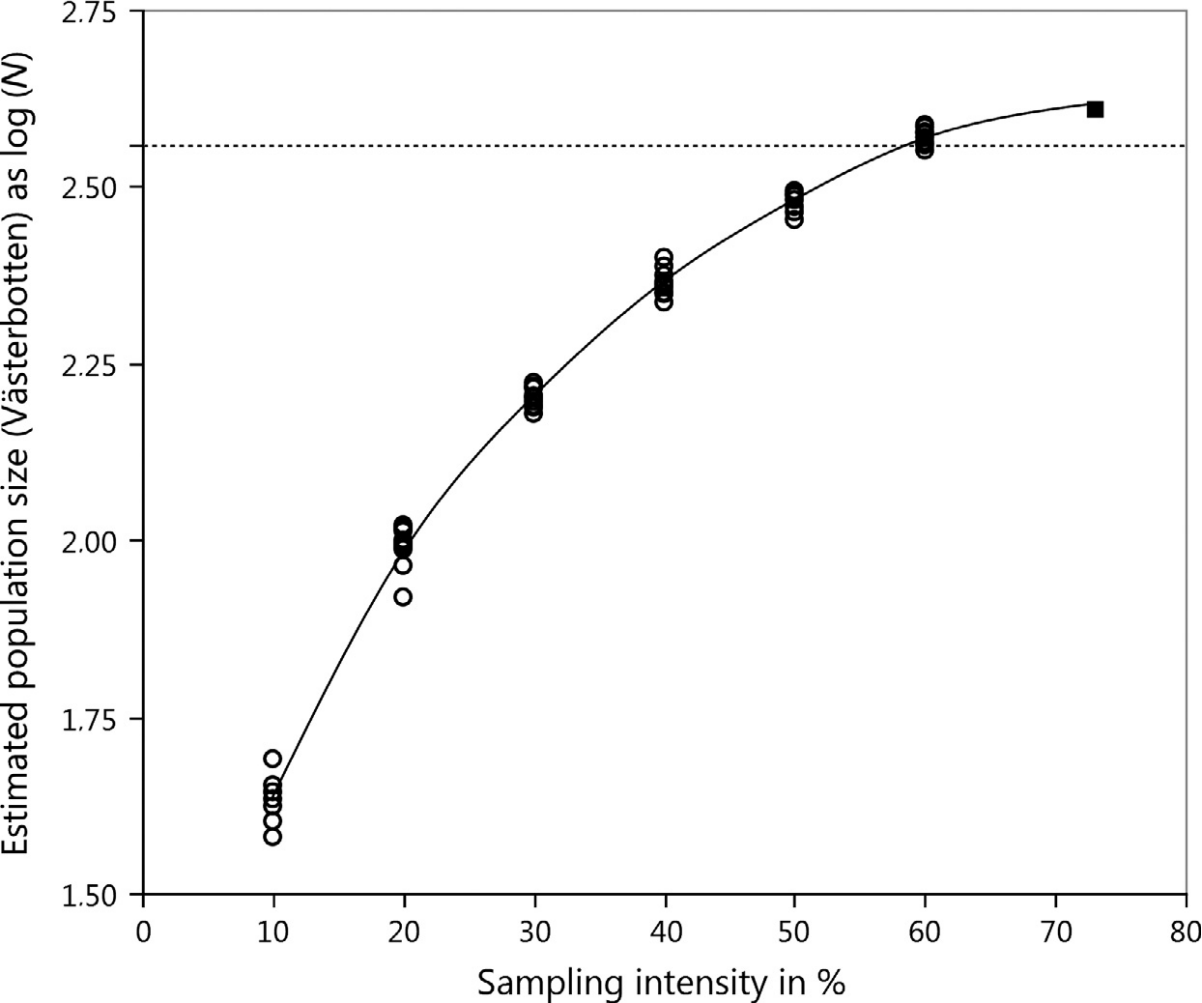
The precision and accuracy of the CRE improved with increasing sampling intensity. The *y*‐axis is on a log scale to show the changes in variance of the population estimates at different sampling intensities (precision) and their distance from the true value (accuracy) in correct proportions. The dashed line denotes the official population estimate for Västerbotten ($\hat{N}$ = 362) which was assumed to be the true population size (100%). The filled square represents the full set of genotypes (*n* = 265) for Västerbotten which corresponds to a sampling intensity of 73%.

Testing for effects of sampling intensity with our empirical data, we found a similar pattern as Creel and Rosenblatt (2013) did in their simulations (Fig. 5). At a sampling intensity of 10%, the coefficient of variation (CV) for the different CRE estimates was 7% and the percentage difference to the official population size estimate 157%; at a sampling intensity of 60%, both CV and percentage difference decreased to 3%. This suggests that CRE population estimates increase in both precision and accuracy with increasing sampling intensity.

Our tests for edge effects of sampling boundaries revealed no significant differences in completeness of the pedigree between the core area and four peripheral border areas in Dalarna–Gävleborg, *χ*²(4)* *=* *7.05, *P *=* *0.134, or Västerbotten, *χ*²(4)* *=* *1.97, *P *=* *0.74.

**Figure 6:**
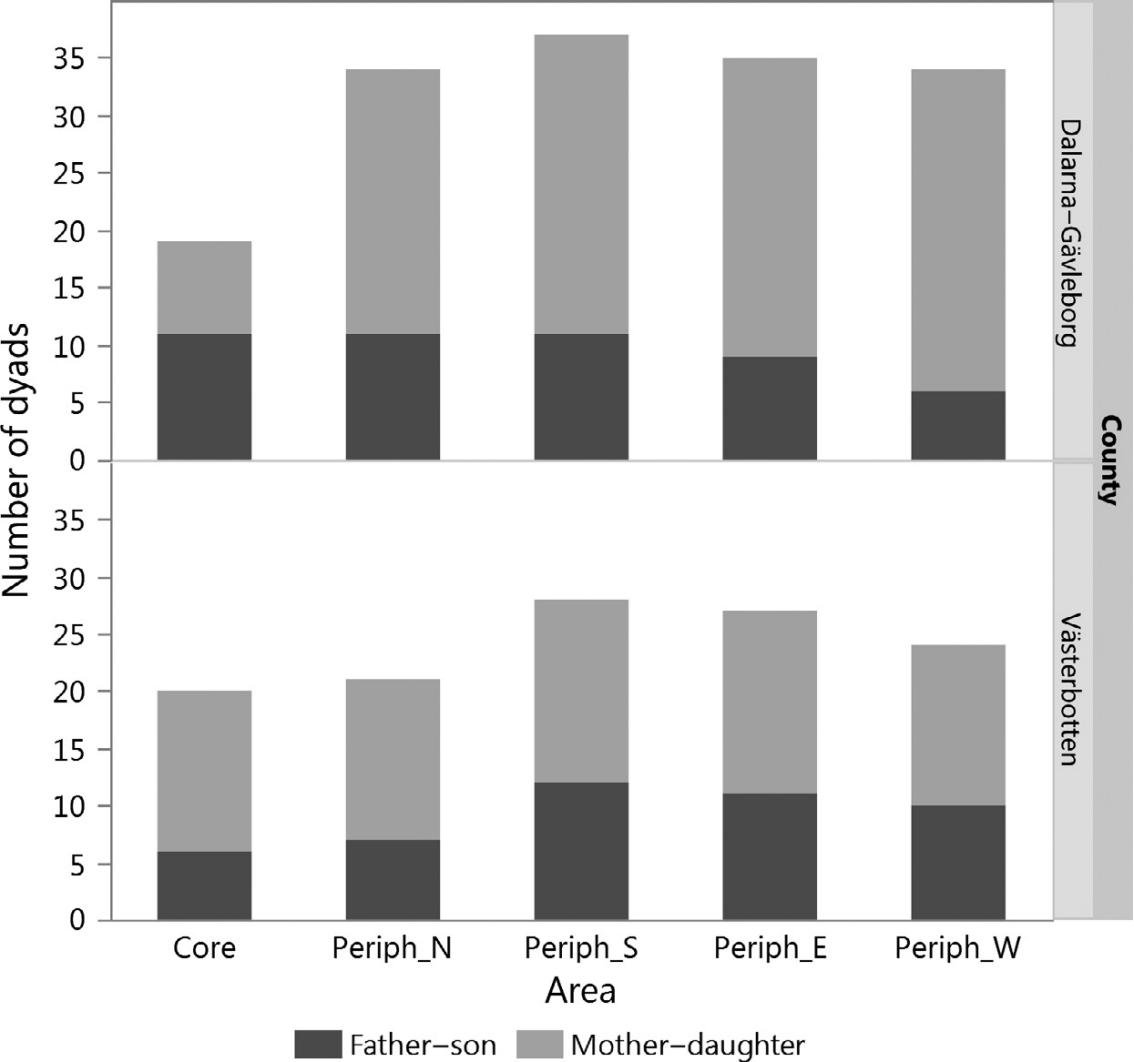
Mother–daughter (light gray) and father–son dyads (dark gray) in the pedigree per sampled individuals (*n* = 50 in Dalarna–Gävleborg, *n* = 30 in Västerbotten) in the core and along the peripheral boundaries of the counties. Except for the core of Dalarna–Gävleborg, there are generally more mother–daughter than father–son dyads in each area, indicating female philopatry.

When males and females were sampled separately, significantly more mother–daughter than father–son dyads were found in Dalarna–Gävleborg, *χ*²(9)* *=* *62.79, *P *<* *0.0001 (Fig. 6). When the same number of males and females (*n* = 50) were sampled randomly across the whole area, there was no significant difference between the proportions of mother–daughter and father–son dyads per sampled individuals, *z* = −0.521, *P *=* *0.602. In Västerbotten, the difference in proportions between male and female dyads was not significant, *χ*²(9)* *=* *15.28, *P *=* *0.083. The two‐sample *z*‐test for proportions when females and males (*n *=* *30) were sampled randomly across the whole area was also not significant, *z* = −0.645, *P *=* *0.52.

## Discussion

In this study, we applied the recently developed Creel–Rosenblatt estimator (CRE), a pedigree reconstruction‐based method, to estimate the size of two fractions of the Swedish brown bear population. SNP genotypes obtained from noninvasively collected fecal samples were used to reconstruct pedigrees from which we were able to infer the presence of additional individuals which otherwise would have remained undetected. Compared to a simple count of detected genotypes, the CRE increased the population estimates by 45% for Dalarna–Gävleborg and 54% for Västerbotten. The circumstance that reliable population estimates were available prior to this study provided an excellent opportunity for testing this new census method because it allowed for the verification of the results. Our pedigree reconstruction‐based population estimates of the CRE fell within the confidence limits of the most recent official estimates. This is an indication that the method provides a potential alternative to traditional CMR approaches with the added benefit that it can be employed using data from a single sampling event. While their percentage relative precision (PRP), a measure which relates a population estimate to its 95% confidence limits (Sutherland 2006), is actually quite good for the study of natural populations (21% in Västerbotten and 35% in Dalarna–Gävleborg), their confidence limits are nevertheless wide. It is therefore difficult to ascertain how close our estimates really are to the true population size. However, the CRE results appear to be further corroborated by the rarefaction results. Simulations by Valière (2002) have shown that the model we used to extrapolate the rarefaction curves has a tendency to overestimate population size if the sampling effort is high. As shown in Figure 4, the rarefaction results indeed exceed both the CMR and CRE estimates and more so in Västerbotten where the sampling intensity was higher. This suggests that the CRE results are actually close to the true figure.

As previously demonstrated by Creel and Rosenblatt (2013), we found that the method works best if the sampling intensity exceeds ~40%. At lower sampling intensities, the estimator tends to severely underestimate the population size. This can be explained by the fact that small sample sizes usually do not contain many parent–offspring pairs which severely restricts the pedigree reconstruction. At much higher sampling intensities (e.g., >80%), hardly any information is gained over a simple count (see Creel and Rosenblatt 2013). Moreover, the risk of overestimation also increases. If, in the extreme case, 100% of individuals were sampled and no reliable information of mortality was available, the CRE would severely overestimate the size of the population because individuals (albeit dead) would still be inferred from the pedigree (Creel and Rosenblatt 2013). The CRE is therefore best suited for noncyclical species with generational overlap and either low or well‐documented mortality rates. The published mortality rates for Swedish brown bears are likely to be accurate because natural mortality of adults is rare in comparison with hunting or traffic accidents which are closely monitored (Mörner et al. 2005). Gross overestimation due to unknown mortality rates can also be avoided by comparing the CRE estimates to those obtained from rarefaction analysis on the same data because the slope and amplitude of the rarefaction curve provide good approximations of population size and the proportion sampled. Contrary to our expectation, we found no significant differences in the completeness of the pedigree when sampling individuals from the peripheries of the study area compared to the center regions. This suggests that many individuals roamed widely with frequent crossings in and out of the study area. Nor did we see a more incomplete pedigree in the west where mountains and low human population density result in a lower sampling effort, suggesting that a similar proportion of individuals are sampled in this area too. Indeed, all peripheral areas were similar, including the hard border to the east, the Baltic Sea. The circumstance that more mother–daughter than father–son dyads were found when separately sampling the same number of individuals from both sexes within a specific area is expected as brown bears show female philopatry (Blanchard and Knight 1991; Støen et al. 2005; Saarma and Kojola 2007).

For well‐studied populations that are regularly sampled, the CRE offers no immediate advantage over established CMR methods in terms of estimating population size. However, if sampling occurs over a number of years, the required sampling effort to maintain a desired sampling coverage should be considerably reduced as genotyped individuals accumulate. In simulations by Creel and Rosenblatt (2013), the proportion of the population that had to be sampled typically dropped to ≤20% within 3 years. The CRE could therefore prove to be useful in situations where budgetary or logistic constraints make repeated, large‐scale sampling events unrealistic, for example, in remote regions or developing countries.

In their simulations, Creel and Rosenblatt (2013) tracked all individuals throughout the simulated period of 15 years which means they consistently had accurate information about parent–offspring relationships and mortalities. Based on these data, they were able to infer individuals (as the missing parent in parent–offspring dyads) without error. In pedigrees reconstructed from empirical genetic field data, this inference is less straightforward especially for species with nonmonogamous mating behavior. Inferring the correct number of missing sires or dams continues to be a major challenge of the CRE method. The additional information provided by the Lynch–Ritland relatedness coefficient (*r*) helped to improve the resolution of the pedigree by enabling us to detect full‐siblings among the parent–offspring dyads which then allowed for correct inference of the missing sire or dam. Based on the values of *r,* we suspect that there are several half‐siblings which share the invisible parent. Unfortunately, it is not sufficient to infer the missing parent as one individual in these cases because the coefficient only captures the degree of relatedness and not the specific relationship. Half‐siblings share on average approximately 25% of alleles but the same is true for grandparent–grandoffspring and avuncular relationships (Blouin 2003). Thus, the true relationship between two individuals can usually not be inferred from their degree of relatedness alone.

To further improve the inferences from the pedigree, information about the age of the sampled individuals is needed. If genetic relatedness can be combined with age in the analysis, the most probable relationships are easily determined. We recommend keeping track of each genotyped individual from the date it was first recorded. Even if the true age remains unknown, a minimum age can be assigned, and over the course of several sampling periods, individuals can at least be compared on the basis of age relative to one another. This would considerably improve the accuracy of the pedigrees, particularly with regard to the directionality in putative parent–offspring dyads (Kopps et al. 2015), thereby helping to refine the CRE population estimates. Using the ratio of known dams and sires for inference of individuals may not fully reflect reality, but given the restrictions of the data, we have shown that it results in a credible estimate.

Concurrent with the simulation results of Creel and Rosenblatt (2013), and using empirical data, we show that accurate estimates of total population size are possible from reconstructed pedigrees. The estimator is limited by the resolution of the pedigree and potentially unknown mortality rates. It therefore works best in long‐lived species with lots of generational overlap and is further helped if “first seen” records are kept to give rough estimates of age. This makes the method particularly appealing for recurring sampling in the same population.

## Conflict of Interest

None declared.

## References

[ece32076-bib-0001] Anderson, E. C. , and J. Garza . 2006 The power of single‐ nucleotide polymorphisms for large‐scale parentage inference. Genetics 172:2567–2582. 16387880 10.1534/genetics.105.048074 PMC1456362

[ece32076-bib-0002] Bellemain, E. , J. E. Swenson , D. Tallmon , P. Taberlet , and S. Brunberg . 2005 Estimating population size of elusive animals with DNA from hunter‐collected feces: four methods for brown bears. Conserv. Biol. 19:150–161.

[ece32076-bib-0003] Blanchard, B. M. , and R. R. Knight . 1991 Movements of Yellowstone grizzly bears. Biol. Conserv. 58:41–67.

[ece32076-bib-0004] Blouin, M. S. 2003 DNA‐ based methods for pedigree reconstruction and kinship analysis in natural populations. Trends Ecol. Evol. 18:503–511.

[ece32076-bib-0005] Brumfield, R. T. , P. Beerli , D. A. Nickerson , and S. V. Edwards . 2003 The utility of single nucleotide polymorphisms in inferences of population history. Trends Ecol. Evol. 18:249–256.

[ece32076-bib-0006] Calboli, F. C. F. , J. Sampson , N. Fretwell , and D. J. Balding . 2008 Population structure and inbreeding from pedigree analysis of purebred dogs. Genetics 179:593. 18493074 10.1534/genetics.107.084954 PMC2390636

[ece32076-bib-0007] Colwell, R. K. , and J. A. Coddington . 1994 Estimating terrestrial biodiversity through extrapolation. Philos. Trans. R. Soc. Lond. B Biol. Sci. 345:101–118. 7972351 10.1098/rstb.1994.0091

[ece32076-bib-0008] Creel, S. 2002 Social Organization and Effective Population Size in Carnivores Pp. 246–269. *in* Caro T. M. (ed.) Behavioral ecology and conservation biology. Chicago, IL: University of Chicago Press.

[ece32076-bib-0009] Creel, S. , and E. Rosenblatt . 2013 Using pedigree reconstruction to estimate population size: genotypes are more than individually unique marks. Ecol. Evol. 3:1294–1304. 23762516 10.1002/ece3.538 PMC3678484

[ece32076-bib-0010] Creel, S. , D. Christianson , S., Liley , and J. A. Winnie . 2007 Predation Risk Affects Reproductive Physiology and Demography of Elk. Science, 315:960. 17303746 10.1126/science.1135918

[ece32076-bib-0011] Csillery, K. , T. Johnson , D. Beraldi , T. Clutton‐Brock , D. Coltman , B. Hansson , et al. 2006 Performance of marker‐ based relatedness estimators in natural populations of outbred vertebrates. Genetics 173:2091–2101. 16783017 10.1534/genetics.106.057331 PMC1569738

[ece32076-bib-0012] Dochtermann, N. , and M. Peacock . 2013 Inter‐ and intra‐specific patterns of density dependence and population size variability in Salmoniformes. Oecologia 171:153–162. 22776906 10.1007/s00442-012-2402-0

[ece32076-bib-0013] ESRI . 2014 ArcGIS desktop: release 10. Environmental Systems Research Institute, Redlands, CA.

[ece32076-bib-0014] Frantzen, M. A. J. , J. B. Silk , J. W. H. Ferguson , R. K. Wayne , and M. H. Kohn . 1998 Empirical evaluation of preservation methods for faecal DNA. Mol. Ecol. 7:1423–1428. 9787450 10.1046/j.1365-294x.1998.00449.x

[ece32076-bib-0015] Hagen, S. B. , and S. G. Aarnes . 2013 Analyserapport. DNA‐analyse av ekskremet‐prøver fra brunbjørn insamlet i Dalarna, Gävleborg och Värmlands län i 2012. Bioforsk, Svanvik, Norway.

[ece32076-bib-0016] Hirata, D. , T. Mano , A. V. Abramov , G. F. Baryshnikov , P. A. Kosintsev , A. A. Vorobiev , et al. 2013 Molecular phylogeography of the brown bear (*Ursus arctos*) in Northeastern Asia based on analyses of complete mitochondrial DNA sequences. Mol. Biol. Evol. 30:1644–1652. 23619144 10.1093/molbev/mst077

[ece32076-bib-0017] Jackson, R. M. , J. D. Roe , R. Wangchuk , and D. O. Hunter . 2006 Estimating snow leopard population abundance using photography and capture‐recapture techniques. Wildl. Soc. Bull. 34:772–781.

[ece32076-bib-0018] Katzner, T. E. , J. A. R. Ivy , E. A. Bragin , E. J. Milner‐Gulland , and J. A. Dewoody . 2011 Conservation implications of inaccurate estimation of cryptic population size. Anim. Conserv. 14:328–332.

[ece32076-bib-0019] Kindberg, J. , and J. E. Swenson . 2013 Beräkning av björnstammens storlek i Värmland, Dalarnas och Gävleborgs län.: Scandinavian Brown Bear Research Project. www.bearproject.info.

[ece32076-bib-0020] Kindberg, J. , and J. E. Swenson . 2015 Björnstammens storlek i Västerbotten 2014.: Scandinavian Brown Bear Research Project. www.bearproject.info.

[ece32076-bib-0021] Kindberg, J. , G. Ericsson , and J. Swenson . 2009 Monitoring rare or elusive large mammals using effort‐corrected voluntary observers. Biol. Conserv. 142:159–165.

[ece32076-bib-0022] Kindberg, J. , J. E. Swenson , G. Ericsson , E. Bellemain , C. Miquel , and P. Taberlet . 2011 Estimating population size and trends of the Swedish brown bear *Ursus arctos* population. Wildl. Biol. 17:114–123.

[ece32076-bib-0023] King, C. M. , D. Purdey , S. A. Davis , and B. Lawrence . 2003 Capture probability and heterogeneity of trap response in stoats (*Mustela erminea*). Wildl. Res. 30:611–619.

[ece32076-bib-0024] Kohn, M. H. , and R. K. Wayne . 1997 Facts from feces revisited. Trends Ecol. Evol. 12:223–227. 21238046 10.1016/s0169-5347(97)01050-1

[ece32076-bib-0025] Kohn, M. H. , R. K. Wayne , E. C. York , D. A. Kamradt , G. Haught , and R. M. Sauvajot . 1999 Estimating population size by genotyping faeces. Proc. R. Soc. Lond. ‐ B. Biol. Sci., 266:657–663. 10.1098/rspb.1999.0686 PMC1689828 10331287

[ece32076-bib-0026] Kojola, I. , P. Helle , S. Heikkinen , H. Lindén , A. Paasivaara , and M. Wikman . 2014 Tracks in snow and population size estimation: the wolf Canis lupus in Finland. Wildl. Biol. 20:279–284.

[ece32076-bib-0027] Kopps, A. M. , J. Kang , W. B. Sherwin , and P. J. Palsböll . 2015 How well do molecular and pedigree relatedness correspond, in populations with diverse mating systems, and various types and quantities of molecular and demographic data?. G3 5:1815–1826. 26134496 10.1534/g3.115.019323 PMC4555218

[ece32076-bib-0028] Korsten, M. , S. Ho , J. Davison , B. Pähn , E. Vulla , M. Roht , et al. 2009 Sudden expansion of a single brown bear maternal lineage across northern continental Eurasia after the last ice age: a general demographic model for mammals? Mol. Ecol. 18:1963–1979. 19434812 10.1111/j.1365-294x.2009.04163.x

[ece32076-bib-0029] Krebs, C. J. 1999 Ecological methodology. Benjamin/Cummings, Menlo Park, CA.

[ece32076-bib-0030] Lande, R. 1988 Genetics and demography in biological conservation. Science 241:1455–1460. 3420403 10.1126/science.3420403

[ece32076-bib-0031] Lewellen, R. H. , and S. H. Vessey . 1998 Modeling biotic and abiotic influences on population size in small mammals. Oecologia 113:210–218. 10.1007/s004420050370 28308199

[ece32076-bib-0032] Logan, K. A. , L. L. Sweanor , J. F. Smith , and M. G. Hornocker . 1999 Capturing pumas with foot‐hold snares. Wildl. Soc. Bull. 27:201–208.

[ece32076-bib-0033] Lynch, M. , and K. Ritland . 1999 Estimation of pairwise relatedness with molecular markers. Genetics 152:1753. 10430599 10.1093/genetics/152.4.1753 PMC1460714

[ece32076-bib-0034] Morin, P. A. , and M. McCarthy . 2007 Highly accurate SNP genotyping from historical and low‐quality samples. Mol. Ecol. Notes 7:937–946.

[ece32076-bib-0035] Morin, P. A. , G. Luikart , R. K. Wayne , and R. K. The SNP Workshop Group . 2004 SNPs in ecology, evolution and conservation. Trends Ecol. Evol., 19:208–216.

[ece32076-bib-0036] Mörner, T. , H. Eriksson , C. Bröjer , K. Nilsson , H. Uhlhorn , E. Agren , et al. 2005 Diseases and mortality in free‐ranging brown bear (*Ursus arctos*), gray wolf (*Canis lupus*), and Wolverine (*Gulo gulo*) in Sweden. J. Wildl. Dis. 41:298–303. 16107663 10.7589/0090-3558-41.2.298

[ece32076-bib-0037] Mowat, G. , B. G. Slough , and R. Rivard . 1994 A comparison of three live capturing devices for lynx: capture efficiency and injuries. Wildl. Soc. Bull. 22:644–650.

[ece32076-bib-0038] Mowry, R. A. , M. E. Gompper , J. Beringer , and L. S. Eggert . 2011 River otter population size estimation using noninvasive latrine surveys. J. Wildl. Manage. 75:1625–1636.

[ece32076-bib-0039] Muñoz‐Igualada, J. , J. A. Shivik , F. G. Domínguez , J. Lara , and L. M. González . 2008 Evaluation of cage‐ traps and cable restraint devices to capture red foxes in Spain. J. Wildl. Manage. 72:830–836.

[ece32076-bib-0040] Nilsson, T. 2013 Population viability analyses of the Scandinavian populations of bear (Ursus arctos) lynx (Lynx lynx) and wolverine (Gulo gulo). Report 6549. Swedish Environmental Protection Agency, Bromma.

[ece32076-bib-0041] Norman, A. J. , and G. Spong . 2015 Single nucleotide polymorphism‐based dispersal estimates using noninvasive sampling. Ecol. Evol. 5:3056–3065. 26357536 10.1002/ece3.1588 PMC4559049

[ece32076-bib-0042] Norman, A. J. , N. R. Street , and G. Spong . 2013 De novo SNP discovery in the Scandinavian brown bear (*Ursus arctos*). PLoS ONE, 8:e81012. 24260529 10.1371/journal.pone.0081012 PMC3832409

[ece32076-bib-0043] Ojaveer, H. , M. Simm , and A. Lankov . 2004 Population dynamics and ecological impact of the non‐indigenous *Cercopagis pengoi* in the Gulf of Riga (Baltic Sea). Int. J. Aquat. Sci. 522:261–269.

[ece32076-bib-0044] Palsboll, P. J. , M. Z. Peery , M. T. Olsen , S. R. Beissinger , and M. Berube . 2013 Inferring recent historic abundance from current genetic diversity. Mol. Ecol. 22:22–40. 23181682 10.1111/mec.12094

[ece32076-bib-0045] Pemberton, J. 2008 Wild pedigrees: the way forward. Proc. R. Soc. B‐Biol. Sci. 275:613–621. 10.1098/rspb.2007.1531 PMC2386891 18211868

[ece32076-bib-0046] Pemberton, J. , S. Albon , F. Guinness , T. Clutton‐Brock , and G. Dover . 1992 Behavioral estimates of male mating success tested by DNA fingerprinting in a polygynous mammal. Behav. Ecol. 3:66–75.

[ece32076-bib-0047] R Development Core Team . 2008 R: a language and environment for statistical computing. R Foundation for Statistical Computing, Vienna, Austria.

[ece32076-bib-0048] Riester, M. , P. F. Stadler , and K. Klemm . 2009 FRANz: reconstruction of wild multi‐generation pedigrees. Bioinformatics 25:2134–2139. 19202194 10.1093/bioinformatics/btp064 PMC2722992

[ece32076-bib-0049] Rolland, J. , M. Basille , É. Marboutin , and J. M. Gaillard . 2011 Comparing profile methods and site‐occupancy modelling for the study of occurrence of an elusive species. Eur. J. Wildl. Res. 57:1115–1118.

[ece32076-bib-0050] Saarma, U. , and I. Kojola . 2007 Matrilineal genetic structure of the brown bear population in Finland. Ursus 18:30–37.

[ece32076-bib-0051] Saarma, U. , S. Y. W. Ho , O. G. Pybus , M. Kaljuste , I. L. Tumanov , I. Kojola , et al. 2007 Mitogenetic structure of brown bears (*Ursus arctos* L.) in northeastern Europe and a new time frame for the formation of European brown bear lineages. Mol. Ecol. 16:401–413. 17217353 10.1111/j.1365-294X.2006.03130.x

[ece32076-bib-0052] SAS Institute, I . JMP Pro version 11.0.0. Cary, NC, 1989‐2007.

[ece32076-bib-0053] Schregel, J. , A. Kopatz , S. B. Hagen , H. Broseth , M. E. Smith , S. Wikan , et al. 2012 Limited gene flow among brown bear populations in far Northern Europe? Genetic analysis of the east‐west border population in the Pasvik Valley. Mol. Ecol. 21:3474–3488. 22680614 10.1111/j.1365-294X.2012.05631.x

[ece32076-bib-0054] Schwartz, M. K. , G. Luikart , and R. S. Waples . 2007 Genetic monitoring as a promising tool for conservation and management. Trends Ecol. Evol. 22:25–33. 16962204 10.1016/j.tree.2006.08.009

[ece32076-bib-0055] Sih, A. , and A. Bell . 2008 Insights for behavioral ecology from behavioral syndromes. Adv. Study Behav. 38:227–281. 24991063 10.1016/S0065-3454(08)00005-3 PMC4075144

[ece32076-bib-0056] Stansbury, C. R. , D. E. Ausband , P. Zager , C. M. Mack , C. R. Miller , M. W. Pennell , et al. 2014 A long‐term population monitoring approach for a wide‐ranging carnivore: noninvasive genetic sampling of gray wolf rendezvous sites in Idaho, USA. J. Wildl. Manage. 78:1040–1049.

[ece32076-bib-0057] Steyaert, S. M. J. G. , A. Endrestøl , K. Hackländer , J. E. Swenson , and A. Zedrosser . 2012 The mating system of the brown bear *Ursus arctos* . Mamm. Rev. 42:12–34.

[ece32076-bib-0058] Støen, O.‐G. , E. Bellemain , S. Sæbø , and J. Swenson . 2005 Kin‐related spatial structure in brown bears *Ursus arctos* . Behav. Ecol. Sociobiol. 59:191–197.

[ece32076-bib-0059] Stone, J. , and M. Björklund . 2001 delrious: a computer program designed to analyse molecular marker data and calculate delta and relatedness estimates with confidence. Mol. Ecol. Notes 1:209–212.

[ece32076-bib-0060] Sugimoto, T. , J. Nagata , V. V. Aramilev , and D. R. McCullough . 2012 Population size estimation of *Amur tigers* in Russian Far East using noninvasive genetic samples. J. Mammal. 93:93–101.

[ece32076-bib-0061] Sunnucks, P. 1998 Avoidance of novel objects by rabbits (*Oryctolagus cuniculus* L.). Wildl. Res. 25:273–283.

[ece32076-bib-0062] Sutherland, W. 2006 Ecological census techniques: a handbook. Cambridge University Press, Cambridge.

[ece32076-bib-0063] Swenson, J. E. , F. Sandegren , S. Brunberg , and P. Segerstrom . 2001 Factors associated with loss of brown bear cubs in Sweden. Ursus 12:69–80.

[ece32076-bib-0064] Swenson, J. E. , P. Taberlet , and E. Bellemain . 2011 Genetics and conservation of European brown bears *Ursus arctos* . Mamm. Rev. 41:87–98.

[ece32076-bib-0065] Taberlet, P. , and J. Bouvet . 1994 Mitochondrial DNA polymorphism, phylogeography, and conservation genetics of the brown bear *Ursus arctos* in Europe. Proc. R. Soc. Lond. ‐ B. Biol. Sci., 255:195–200. 10.1098/rspb.1994.0028 8022838

[ece32076-bib-0066] Taberlet, P. , J. E. Swenson , F. Sandegren , and A. Bjarvall . 1995 Localization of a contact zone between two highly divergent mitochondrial DNA lineages of the brown bear *Ursus arctos* in Scandinavia. Conserv. Biol. 9:1255–1261. 10.1046/j.1523-1739.1995.951255.x 34261288

[ece32076-bib-0067] Taberlet, P. , G. Luikart , and L. P. Waits . 1999 Noninvasive genetic sampling: look before you leap. Trends Ecol. Evol. 14:323–327. 10407432 10.1016/s0169-5347(99)01637-7

[ece32076-bib-0068] Tuyttens, F. A. M. , D. W. Macdonald , C. Newman , R. Delahay , L. M. Rogers , F. J. Mallinson , et al. 1999 Differences in trappability of European badgers *Meles meles* in three populations in England. J. Appl. Ecol. 36:1051–1062.

[ece32076-bib-0069] Valderrama, S. V. , L. E. Molles , and J. R. Waas . 2013 Effects of population size on singing behavior of a rare duetting songbird. Conserv. Biol. 27:210–218. 22979901 10.1111/j.1523-1739.2012.01917.x

[ece32076-bib-0070] Valière, N. 2002 gimlet: a computer program for analysing genetic individual identification data. Mol. Ecol. Notes 2:377–379.

[ece32076-bib-0071] Vié, J.‐C. , C. Hilton‐Taylor , and S. N. Stuart . 2009 Wildlife in a changing world; IUCN red list of threatened species. 2008: an analysis of the 2008 IUCN red list of threatened species. Analysis of the 2008 IUCN red list of threatened species. Gland, Switzerland.

[ece32076-bib-0072] Waits, L. P. , and D. Paetkau . 2005 Noninvasive genetic sampling tools for wildlife biologists: a review of applications and recommendations for accurate data collection. J. Wildl. Manage. 69:1419–1433.

[ece32076-bib-0073] Waits, L. , P. Taberlet , J. E. Swenson , F. Sandegren , and R. Franzen . 2000 Nuclear DNA microsatellite analysis of genetic diversity and gene flow in the Scandinavian brown bear (*Ursus arctos*). Mol. Ecol. 9:421–431. 10736045 10.1046/j.1365-294x.2000.00892.x

[ece32076-bib-0074] Wang, J. 2011 COANCESTRY: a program for simulating, estimating and analysing relatedness and inbreeding coefficients. Mol. Ecol. Resour. 11:141–145. 21429111 10.1111/j.1755-0998.2010.02885.x

[ece32076-bib-0075] Wilson, G. J. , and R. J. Delahay . 2001 A review of methods to estimate the abundance of terrestrial carnivores using field signs and observation. Wildl. Res. 28:151–164.

